# *N*′-[1-(4-Methoxy­phen­yl)ethyl­idene]acetohydrazide

**DOI:** 10.1107/S1600536808037677

**Published:** 2008-11-22

**Authors:** Yu-Feng Li, Fang-Fang Jian

**Affiliations:** a Microscale Science Institute, Weifang University, Weifang 261061, People’s Republic of China

## Abstract

The title compound, C_11_H_14_N_2_O_2_, was prepared by the reaction of acetohydrazide and 1-(4-methoxy­phen­yl)ethanone. In the mol­ecule, all bond lengths and angles are within normal ranges. In the crystal structure, adjacent mol­ecules are linked into a centrosymmetric dimer by inter­molecular N—H⋯O hydrogen bonding.

## Related literature

For related literature, see: Cimerman *et al.* (1997 ▶); Girgis (2006 ▶). For bond-length data, see: Allen *et al.* (1987 ▶).
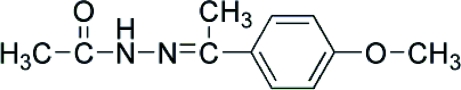


## Experimental

### 

#### Crystal data

C_11_H_14_N_2_O_2_
*M* *_r_* = 206.24
Monoclinic, 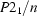

*a* = 13.282 (3) Å
*b* = 4.9923 (10) Å
*c* = 16.854 (3) Å
β = 98.88 (3)°
*V* = 1104.2 (4) Å^3^
*Z* = 4
Mo *K*α radiation
μ = 0.09 mm^−1^
*T* = 293 (2) K
0.25 × 0.20 × 0.18 mm

#### Data collection

Bruker SMART CCD area-detector diffractometer
Absorption correction: none
6830 measured reflections
2681 independent reflections
1228 reflections with *I* > 2σ(*I*)
*R*_int_ = 0.035

#### Refinement

*R*[*F* ^2^ > 2σ(*F* ^2^)] = 0.058
*wR*(*F* ^2^) = 0.196
*S* = 0.93
2681 reflections
137 parameters
H-atom parameters constrained
Δρ_max_ = 0.17 e Å^−3^
Δρ_min_ = −0.17 e Å^−3^

### 

Data collection: *SMART* (Bruker, 1997 ▶); cell refinement: *SAINT* (Bruker, 1997 ▶); data reduction: *SAINT*; program(s) used to solve structure: *SHELXS97* (Sheldrick, 2008 ▶); program(s) used to refine structure: *SHELXL97* (Sheldrick, 2008 ▶); molecular graphics: *SHELXTL* (Sheldrick, 2008 ▶); software used to prepare material for publication: *SHELXTL*.

## Supplementary Material

Crystal structure: contains datablocks global, I. DOI: 10.1107/S1600536808037677/at2657sup1.cif

Structure factors: contains datablocks I. DOI: 10.1107/S1600536808037677/at2657Isup2.hkl

Additional supplementary materials:  crystallographic information; 3D view; checkCIF report

## Figures and Tables

**Table 1:** Hydrogen-bond geometry (Å, °)

*D*—H⋯*A*	*D*—H	H⋯*A*	*D*⋯*A*	*D*—H⋯*A*
N2—H2*A*⋯O2^i^	0.86	2.12	2.956 (3)	166

Symmetry code: (i) 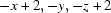.

**Fig. 1.**
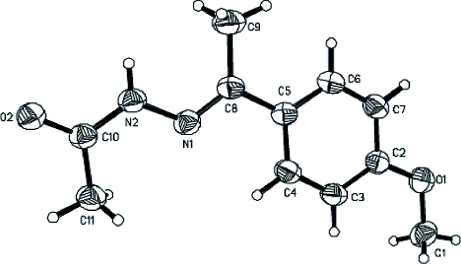
The structure of the title compound showing 30% probability displacement ellipsoids and the atom-numbering scheme.

**Fig. 2.**
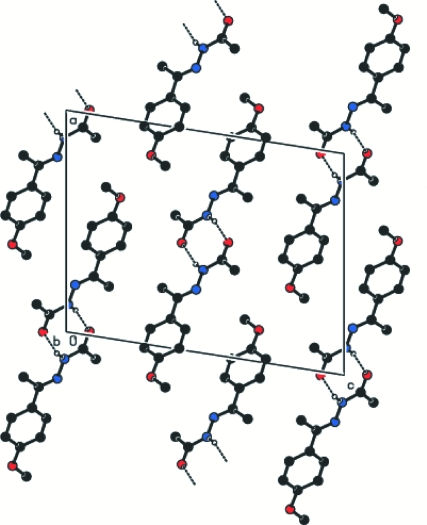
Part of the crystal structure of the title compound showing the formation of a cyclic centrosymmetric dimer. For the sake of clarity, H atoms not involved in hydrogen bonding have been omitted.

## supplementary crystallographic information

### Comment

Schiff bases have received considerable attention in the literature. They are attractive from several points of view, such as the possibility of analytical application (Cimerman *et al.*, 1997). As part of our search for new Schiff base compounds we synthesized the title compound (I), and describe its structure here.

In the title compound (Fig. 1), all bond lengths and angles are within normal ranges (Allen *et al.*, 1987). The C8—N1 bond length of 1.278 (3)Å is comparable with C—N double bond [1.281 (2) Å] reported (Girgis, 2006).

In the crystal structure, adjacent molecules are linked into a centro-symmetric supra-molecular dimer by intermolecular N—H···O hydrogen bonding (Table 1, Fig. 2).

### Experimental

A mixture of the acetohydrazide (0.1 mol), and 1-(4-methoxyphenyl)ethanone (0.1 mol) was stirred in refluxing ethanol (20 mL) for 4 h to afford the title compound (0.080 mol, yield 80%). Single crystals suitable for X-ray measurements were obtained by recrystallization from ethanol at room temperature.

### Refinement

H atoms were fixed geometrically and allowed to ride on their attached atoms, with C—H = 0.93-0.96 Å and N—H = 0.86 Å, and with *U*_iso_=1.2–1.5*U*_eq_.

### Crystal data

**Table:**

C_11_H_14_N_2_O_2_	*F*_000_ = 440
*M**_r_* = 206.24	*D*_x_ = 1.241 Mg m^−^^3^
Monoclinic, *P*2_1_/*n*	Mo *K*α radiation λ = 0.71073 Å
Hall symbol: -P 2yn	Cell parameters from 831 reflections
*a* = 13.282 (3) Å	θ = 2.4–24.0º
*b* = 4.9923 (10) Å	µ = 0.09 mm^−^^1^
*c* = 16.854 (3) Å	*T* = 293 (2) K
β = 98.88 (3)º	Block, yellow
*V* = 1104.2 (4) Å^3^	0.25 × 0.20 × 0.18 mm
*Z* = 4	

### Data collection

**Table:**

Bruker SMART CCD area-detector diffractometer	1228 reflections with *I* > 2σ(*I*)
Radiation source: fine-focus sealed tube	*R*_int_ = 0.035
Monochromator: graphite	θ_max_ = 28.3º
*T* = 273(2) K	θ_min_ = 1.8º
φ and ω scans	*h* = −17→15
Absorption correction: none	*k* = −6→6
6830 measured reflections	*l* = −16→22
2681 independent reflections	

### Refinement

**Table:**

Refinement on *F*^2^	Secondary atom site location: difference Fourier map
Least-squares matrix: full	Hydrogen site location: inferred from neighbouring sites
*R*[*F*^2^ > 2σ(*F*^2^)] = 0.058	H-atom parameters constrained
*wR*(*F*^2^) = 0.196	*w* = 1/[σ^2^(*F*_o_^2^) + (0.0986*P*)^2^ + 0.0719*P*] where *P* = (*F*_o_^2^ + 2*F*_c_^2^)/3
*S* = 0.93	(Δ/σ)_max_ < 0.001
2681 reflections	Δρ_max_ = 0.17 e Å^−^^3^
137 parameters	Δρ_min_ = −0.17 e Å^−^^3^
Primary atom site location: structure-invariant direct methods	Extinction correction: none

### Special details

**Table:**

Geometry. All e.s.d.'s (except the e.s.d. in the dihedral angle between two l.s. planes) are estimated using the full covariance matrix. The cell e.s.d.'s are taken into account individually in the estimation of e.s.d.'s in distances, angles and torsion angles; correlations between e.s.d.'s in cell parameters are only used when they are defined by crystal symmetry. An approximate (isotropic) treatment of cell e.s.d.'s is used for estimating e.s.d.'s involving l.s. planes.
Refinement. Refinement of *F*^2^ against ALL reflections. The weighted *R*-factor *wR* and goodness of fit *S* are based on *F*^2^, conventional *R*-factors *R* are based on *F*, with *F* set to zero for negative *F*^2^. The threshold expression of *F*^2^ > σ(*F*^2^) is used only for calculating *R*-factors(gt) *etc*. and is not relevant to the choice of reflections for refinement. *R*-factors based on *F*^2^ are statistically about twice as large as those based on *F*, and *R*- factors based on ALL data will be even larger.

### Fractional atomic coordinates and isotropic or equivalent isotropic displacement parameters (Å^2^)

**Table:**

	*x*	*y*	*z*	*U*_iso_*/*U*_eq_	
O2	0.98272 (13)	0.2016 (4)	0.91643 (10)	0.0761 (6)	
O1	1.64324 (12)	0.8045 (4)	1.19345 (10)	0.0788 (6)	
N2	1.12429 (14)	0.2219 (4)	1.00666 (11)	0.0631 (6)	
H2A	1.1037	0.0925	1.0338	0.076*	
N1	1.21780 (14)	0.3415 (4)	1.03226 (11)	0.0608 (5)	
C5	1.36778 (17)	0.4053 (4)	1.12310 (13)	0.0559 (6)	
C10	1.06577 (19)	0.3085 (5)	0.93934 (14)	0.0606 (6)	
C8	1.26860 (17)	0.2683 (4)	1.09940 (13)	0.0572 (6)	
C2	1.55227 (16)	0.6811 (5)	1.16686 (13)	0.0588 (6)	
C7	1.51710 (18)	0.5048 (5)	1.21885 (14)	0.0675 (7)	
H7A	1.5547	0.4775	1.2695	0.081*	
C3	1.49509 (19)	0.7215 (5)	1.09293 (15)	0.0739 (8)	
H3A	1.5174	0.8408	1.0570	0.089*	
C4	1.40476 (19)	0.5860 (6)	1.07177 (14)	0.0752 (8)	
H4A	1.3671	0.6163	1.0213	0.090*	
C6	1.42740 (18)	0.3683 (5)	1.19720 (13)	0.0632 (7)	
H6A	1.4061	0.2477	1.2333	0.076*	
C11	1.1030 (2)	0.5331 (5)	0.89359 (15)	0.0754 (7)	
H11A	1.0532	0.5729	0.8475	0.113*	
H11B	1.1660	0.4828	0.8764	0.113*	
H11C	1.1137	0.6886	0.9273	0.113*	
C9	1.2343 (2)	0.0599 (6)	1.15380 (17)	0.0931 (10)	
H9A	1.1689	0.1090	1.1668	0.159 (16)*	
H9B	1.2828	0.0482	1.2022	0.239*	
H9C	1.2292	−0.1105	1.1271	0.239*	
C1	1.68123 (19)	0.9957 (5)	1.14314 (16)	0.0807 (8)	
H1B	1.7453	1.0639	1.1695	0.121*	
H1C	1.6335	1.1404	1.1324	0.121*	
H1D	1.6906	0.9120	1.0935	0.121*	

### Atomic displacement parameters (Å^2^)

**Table:**

	*U*^11^	*U*^22^	*U*^33^	*U*^12^	*U*^13^	*U*^23^
O2	0.0790 (12)	0.0801 (12)	0.0670 (11)	−0.0227 (10)	0.0048 (9)	0.0087 (9)
O1	0.0687 (11)	0.0908 (13)	0.0735 (12)	−0.0172 (10)	0.0003 (9)	0.0073 (10)
N2	0.0729 (13)	0.0604 (12)	0.0562 (12)	−0.0161 (10)	0.0109 (10)	0.0038 (9)
N1	0.0651 (12)	0.0615 (12)	0.0562 (12)	−0.0126 (9)	0.0107 (9)	−0.0025 (9)
C5	0.0640 (13)	0.0541 (13)	0.0515 (13)	−0.0032 (11)	0.0153 (11)	−0.0005 (10)
C10	0.0718 (16)	0.0557 (14)	0.0558 (14)	−0.0084 (12)	0.0145 (12)	−0.0020 (11)
C8	0.0674 (15)	0.0560 (14)	0.0506 (13)	−0.0047 (12)	0.0162 (11)	−0.0009 (11)
C2	0.0598 (14)	0.0620 (14)	0.0549 (13)	0.0000 (11)	0.0097 (11)	0.0000 (11)
C7	0.0722 (15)	0.0773 (16)	0.0510 (13)	−0.0031 (14)	0.0033 (11)	0.0059 (12)
C3	0.0746 (16)	0.0863 (19)	0.0598 (15)	−0.0177 (15)	0.0072 (13)	0.0196 (14)
C4	0.0759 (16)	0.0917 (19)	0.0542 (14)	−0.0197 (15)	−0.0016 (12)	0.0178 (13)
C6	0.0768 (16)	0.0603 (14)	0.0533 (14)	−0.0024 (12)	0.0130 (12)	0.0081 (11)
C11	0.0877 (17)	0.0683 (16)	0.0696 (16)	−0.0160 (14)	0.0099 (13)	0.0132 (13)
C9	0.101 (2)	0.097 (2)	0.0798 (19)	−0.0350 (18)	0.0105 (16)	0.0259 (17)
C1	0.0726 (16)	0.0842 (19)	0.0872 (18)	−0.0190 (15)	0.0183 (14)	0.0019 (16)

### Geometric parameters (Å, °)

**Table:**

O2—C10	1.232 (3)	C7—H7A	0.9300
O1—C2	1.369 (3)	C3—C4	1.376 (3)
O1—C1	1.420 (3)	C3—H3A	0.9300
N2—C10	1.344 (3)	C4—H4A	0.9300
N2—N1	1.386 (2)	C6—H6A	0.9300
N2—H2A	0.8600	C11—H11A	0.9600
N1—C8	1.278 (3)	C11—H11B	0.9600
C5—C6	1.384 (3)	C11—H11C	0.9600
C5—C4	1.391 (3)	C9—H9A	0.9600
C5—C8	1.483 (3)	C9—H9B	0.9600
C10—C11	1.488 (3)	C9—H9C	0.9600
C8—C9	1.503 (3)	C1—H1B	0.9600
C2—C3	1.370 (3)	C1—H1C	0.9600
C2—C7	1.374 (3)	C1—H1D	0.9600
C7—C6	1.372 (3)		
			
C2—O1—C1	118.96 (19)	C3—C4—H4A	118.8
C10—N2—N1	119.9 (2)	C5—C4—H4A	118.8
C10—N2—H2A	120.1	C7—C6—C5	121.7 (2)
N1—N2—H2A	120.1	C7—C6—H6A	119.1
C8—N1—N2	118.54 (19)	C5—C6—H6A	119.1
C6—C5—C4	116.1 (2)	C10—C11—H11A	109.5
C6—C5—C8	122.8 (2)	C10—C11—H11B	109.5
C4—C5—C8	121.0 (2)	H11A—C11—H11B	109.5
O2—C10—N2	119.9 (2)	C10—C11—H11C	109.5
O2—C10—C11	121.1 (2)	H11A—C11—H11C	109.5
N2—C10—C11	119.0 (2)	H11B—C11—H11C	109.5
N1—C8—C5	115.6 (2)	C8—C9—H9A	109.5
N1—C8—C9	124.7 (2)	C8—C9—H9B	109.5
C5—C8—C9	119.7 (2)	H9A—C9—H9B	109.5
O1—C2—C3	124.7 (2)	C8—C9—H9C	109.5
O1—C2—C7	116.7 (2)	H9A—C9—H9C	109.5
C3—C2—C7	118.6 (2)	H9B—C9—H9C	109.5
C6—C7—C2	121.0 (2)	O1—C1—H1B	109.5
C6—C7—H7A	119.5	O1—C1—H1C	109.5
C2—C7—H7A	119.5	H1B—C1—H1C	109.5
C2—C3—C4	120.1 (2)	O1—C1—H1D	109.5
C2—C3—H3A	119.9	H1B—C1—H1D	109.5
C4—C3—H3A	119.9	H1C—C1—H1D	109.5
C3—C4—C5	122.4 (2)		

### Hydrogen-bond geometry (Å, °)

**Table:**

*D*—H···*A*	*D*—H	H···*A*	*D*···*A*	*D*—H···*A*
N2—H2A···O2^i^	0.86	2.12	2.956 (3)	166
C4—H4A···N1	0.93	2.44	2.755 (3)	100

Symmetry codes: (i) −*x*+2, −*y*, −*z*+2.
